# Repair or replace the mitral valve for severe ischemic mitral regurgitation?

**DOI:** 10.1186/1749-8090-8-S1-O213

**Published:** 2013-09-11

**Authors:** V Shumavets, A Shket, A Janushko, V Sevrukevich, I Grinchuk, S Kurganovich, N Semenova, Y Ostrovski

**Affiliations:** 1 Cardiac Surgery Department, Belarus Cardiology Centre, Minsk, Belarus

## Background

We focused our study on patients with severe compromised ischemic left ventricle and functional mitral regurgitation to understand how mitral valve replacement versus repair affects survival and provide the predictors of hospital and long-term mortality.

## Methods

870 patients (mean age, 57,9±8,3 years) from 2000 to 2012, with coronary artery diseases and significant ischemic mitral regurgitation (>2) were operated – in 787pts CABG+MV repair were performed and in 83 pts MV replacement were combined with CABG. Groups were matched by propensity score using demographics dates, co-morbidity, LV remodeling and MR grade by quantitative echocardiography. Survival (with mean follow-up 5,3±1,6 years) and NYHA functional class were compared. The impact of mitral valve replacement versus repair on survival by comparing theses propensity matched subgroups was analyzed.

## Results

Follow-up was 100% complete. Before matching 10-year survival was significantly worse in replacement group (long-rank p=0,003). After propensity matching 1:2 we’ve received homogenous cohort of 99 pts with severe compromised LV (EDD 71,8±7,6 mm, ESD 59±8,1 mm, iEDV 131±28,1 ml/m2 with EF 31,3±3,7%) and severe MR. The fact of mitral valve replacement versus repair did not influence survival (long-rank p=0,443) and overall in 1- and 5-year it were 91,8±0,14% and 69,2±0,48% respectively. The independent risk factors for an increased mortality within the five years of surgery in multivariate propensity-matched analyses were found LV ESD (HR–1,085, 95% CI 1,018–1,157, p=0,013), chronic renal disease (HR–1,8, 95% CI 1,34–2,45 p=0,012), use of IABP during hospital period (HR–3,147, 95% CI 1,17–8,4, p=0,022) and age (HR–0,936, 95% CI 0,876–1,0, p=0,011).

## Conclusion

The mitral valve replacement versus repair did not seem to affect survival in patients with severe damaged ischemic LV and it mostly depends on factors related to the patient’s condition at the time of surgery.

